# Empirical analysis of phase-amplitude coupling approaches

**DOI:** 10.1371/journal.pone.0219264

**Published:** 2019-07-09

**Authors:** Michael Caiola, Annaelle Devergnas, Mark H. Holmes, Thomas Wichmann

**Affiliations:** 1 Yerkes National Primate Research Center, Emory University, Atlanta, GA, United States of America; 2 Udall Center of Excellence for Parkinson’s Disease Research at Emory University, Atlanta, GA, United States of America; 3 Department of Neurology, School of Medicine, Emory University, Atlanta, GA, United States of America; 4 Department of Mathematical Sciences, Rensselaer Polytechnic Institute, Troy, NY, United States of America; University of British Columbia, CANADA

## Abstract

Analysis of the coupling between the phases and amplitudes of oscillations within the same continuously sampled signal has provided interesting insights into the physiology of memory and other brain process, and, more recently, the pathophysiology of parkinsonism and other movement disorders. Technical aspects of the analysis have a significant impact on the results. We present an empirical exploration of a variety of analysis design choices that need to be considered when measuring phase-amplitude coupling (PAC). We studied three alternative filtering approaches to the commonly used Kullback–Leibler distance-based method of PAC analysis, including one method that uses wavelets, one that uses constant filter settings, and one in which filtering of the data is optimized for individual frequency bands. Additionally, we introduce a time-dependent PAC analysis technique that takes advantage of the inherent temporality of wavelets. We examined how the duration of the sampled data, the stability of oscillations, or the presence of artifacts affect the value of the “modulation index”, a commonly used parameter describing the degree of PAC. We also studied the computational costs associated with calculating modulation indices by the three techniques. We found that wavelet-based PAC performs better with similar or less computational cost than the two other methods while also allowing to examine temporal changes of PAC. We also show that the reliability of PAC measurements strongly depends on the duration and stability of PAC, and the presence (or absence) of artifacts. The best parameters to be used for PAC analyses of long samples of data may differ, depending on data characteristics and analysis objectives. Prior to settling on a specific PAC analysis approach for a given set of data, it may be useful to conduct an initial analysis of the time-dependence of PAC using our time-resolved PAC analysis.

## Introduction

There is substantial interest in new methods to analyze local field potentials (LFPs) or electroencephalography/electrocorticogram (EEG/ECoG) signals in normal and disease states [1–6]. Traditionally, such analyses have consisted of studies of power spectral characteristics of the data, but recent reports have emphasized additional analysis approaches, specifically studies of the coupling between the phase of low-frequency components of such signals and the amplitude of higher frequency components [1–4]. Practical use of these analyses is exemplified by the fact that the expression of specific patterns of phase-amplitude coupling (PAC) within cortical EEG, ECoG signals, or in LFP recordings from the basal ganglia has been discussed as a possible biomarker of disease severity in patients with Parkinson’s disease or dystonia [3, 7]. A commonly used method to quantify PAC is the calculation of “modulation indices” (MIs) at pairings of specific amplitude and phase frequency ranges [2, 3, 7–14]. Most investigators prefer a type of MI calculation that uses the entropy-based Kullback–Leibler (KL) distance for its analysis. KL-based MI calculation is independent of the average size of the input signal, tolerant to noise, distinguishes between multimodal signals, and was shown to outperform other methods [15]. The KL distance assigns a score ranging from 0 (no coupling) to 1 (complete coupling) to the degree of clustering of the angular distribution of specific spectral components of the signal [16]. Since its implementation, several variants of KL distance-based MI calculations have been developed. In this study, we compare results generated with the conventional method to those generated with two of these variants, one that preserves temporality with wavelets and one that adjusts its filtering bandwidth based on phase frequencies. According to our analysis, both variants show improvements over the original in terms of frequency resolution and the accuracy of PAC detection. We also examined the dependence of the MI measures on filtering approaches, the length of data, the signal to noise ratio (SNR) of the data, and the simultaneous or successive presence of multiple PAC targets in the input data. Finally, we analyzed the computational cost of determining the MI with the different filtering methods, and present a novel method of visualization of time-evolving changes of PAC.

## Materials and methods

### General approach

We generated color-coded maps (comodulograms) depicting the strength of the coupling between the amplitude and the phase of frequencies within data that were synthetically generated to represent specific data characteristics such as a defined SNR, or (known) PAC at specific frequencies. MI values were used as a measure of PAC. All analyses were carried out with custom-written MATLAB scripts (MATLAB 9.3; MathWorks, Natick, MA, USA), using parallelized code running on up to four CPU cores. In other analyses, the use of the various methods of PAC analysis in “real” cortical local field potential data was also explored.

### Generation of synthetic “data”

We generated synthetic LFP-like signals [15, 17] that contained phase-amplitude coupled oscillations for part or all of the record, using a superposition of three waves, i.e., one sine wave at a slow “phase” frequency, another sine wave at a faster “amplitude” frequency, and a third, consisting of a multiplication of the previous two sine waves:

signal=sin(2πphase)+0.2sin(2πamplitude)+0.2sin(2πphase)sin(2πamplitude).

Since sin(*a*)sin(*b*) = .5(cos(*a* − *b*) − cos(*a* + *b*)), the synthetic signal has spectral peaks at the phase and amplitude frequencies as well as smaller spectral “sidebands” at phase ± amplitude. This superposition was then added to a normally distributed noise signal (Gaussian white noise) and sampled at 1000 Hz. If the phase frequency is close to (or within) the filter envelope for the amplitude frequency, false peaks in PAC detection may result. We therefore added a dotted white line to the comodulograms to represent the region of the comodulogram (Amplitude Frequency < 2 × Phase Frequency) where such false interpretations may occur (an example is shown in S1 Fig).

The SNR was calculated as $10\log_{10}\frac{P(\text{signal})}{P(\text{noise})}$, where *P*(*x*) is the average power of *x*, unless otherwise denoted. To generate a particular SNR, the random noise added to the synthetic signal was multiplied by a factor that follows from the previous equation:

$$\frac{-\text{signal}\cdot \text{noise}-\sqrt{{\left(\text{signal}\cdot \text{noise}\right)}^{2}-\left(1-10^{\frac{SNR}{10}}\right){\left\|noise\right\|}^{2}{\left\|signal\right\|}^{2}}}{\left(1-10^{\frac{SNR}{10}}\right){\left\|noise\right\|}^{2}}$$

### Collection of cortical Local Field Potential data in a primate

Spontaneous local field potentials (LFPs) were recorded from an awake nonhuman Rhesus monkey (male, 6kg at the time of recording). The experiments in this animal occurred in the context of another study focused on the pathophysiology of primate parkinsonism and are only described very briefly here. These studies were approved by the Institutional Animal Care and Use Committee of Emory University. The animal originated from the breeding colony at the Yerkes National Primate Research Center. After habituation to the laboratory environment, the animal underwent a surgical procedure under isoflurane anesthesia during which a metal recording chamber was placed on the animal’s skull to give us access to the primary motor cortex (M1). A head fixation bolt was also placed on the skull. Subsequent to the surgery, the animal was chronically treated with weekly injections of the dopaminergic neurotoxin 1-methyl-4-phenyl-1,2,3,6-tetrahydropyridine (MPTP), to induce parkinsonism. Once stable moderate parkinsonism was documented, the animal underwent recording studies aimed at recording M1 LFP signals. These recordings were carried out with a 16-contact linear array electrode (NeuroNexus, Ann Arbor, MI) which was acutely placed into M1, and positioned to allow recordings from deep cortical layers. The resulting signals were band-pass filtered (.1 Hz–700 Hz) [7] and amplified (model 3600 amplifier, AM systems, Sequim, WA), and then sampled at 20000 Hz (Power 1401, CED, Cambridge, UK). Off-line, the signal was down-sampled to a sampling rate of 1000 Hz before PAC analysis.

### Target generation

Phase/amplitude “targets” were randomly selected for the synthetic signals with a generator of uniformly distributed random numbers (MATLAB’s rand function) that guaranteed that the “phase” target (within the range of 0−50 Hz), was smaller than the “amplitude” target (within the range of 0−400 Hz), and that the duration of time during which the PAC target was present exceeded 20 s. These signal ranges were chosen to cover a large portion of the range of frequencies that would typically be found in neural signals. Similar ranges have been used in previous studies [2]. To construct temporal breaks between relevant targets in individual records, targets outside of the analyzed PAC range (greater than 50 Hz for the phase frequency, and 400 Hz for the amplitude frequency) were inserted between targets within the analyzed range. When multiple targets were used, they followed one after another without overlap. For dynamic signals, we took a 5-minute signal of white noise and randomly placed 1 s periods representing a PAC target (coupling of 20 Hz phase frequency and 130 Hz amplitude frequency). For this, the white noise was set to have half the amplitude of the synthetic signal. Spectrograms were constructed using MATLAB’s pwelch function, using Hamming windows, 50% overlap and the default NFFT value (256) and then scaled to clearly show the signal against the background.

### Calculation of MI

For the calculation of KL-distance based MI values (using an algorithm described by Tort et al. [15]), the data were first mean-corrected. The resulting signal, x(t), was then separately band-pass filtered to generate phase and amplitude signals using one of three methods of filtering, the “Conventional Method” (CM), the “Variable Method” (VM), and the “Wavelet Method” (WM). These filters were implemented to not induce phase-shifts. In all cases, the desired bandwidth of the comodulogram is set by the user but the actual filter width is determined by the filtering method.

#### Conventional method

For the CM, we used a method described in Ref. [18] that utilizes a filtering function developed by UCSD’s EEGLAB [19]. Since the publication of this method, EEGLAB has updated this function, so we utilize the current “pop_eegfiltnew” function in place of the original filtering function used by Tort et al. [18]. To generate phase data, the signal was bandpass-filtered around prescribed center frequencies, 4 to 52 Hz, with a bandwidth of 4 Hz every 2 Hz, resulting in *i* = 1,…,25 filtered phase signals (each with 50% overlap), $x_{i}^{f_{\theta }}$, where *f*_*θ*_ represents phase filtering. Similarly, the amplitude signals, $x_{j}^{f_{A}}$, used bandwidths of 10 Hz every 5 Hz, with filter range centers between 15 Hz and 400 Hz, resulting in *j* = 1,…,78 filtered amplitude signals (each with 50% overlap). Other methods of bin creation and overlap were not explored in this study. For each of the prescribed bandwidths, [*a*, *b*], pop_eegfiltnew uses a sinc type I linear phase finite impulse response (FIR) filter with Hamming windows with an (empirically-defined) filter width to be $\left[a-\frac{a}{4},\;b+\frac{a}{4}\right]$ with order $\frac{6.6f_{s}}{a}$, and sampling rate, *f*_*s*_. The ranges and bandwidths can be adjusted with smaller phase bandwidths, leading to a PAC analysis with slightly higher frequency resolution (S2A and S2D Fig).

#### Variable method

For the VM, we used the same center frequencies as were used for the CM (4−52 Hz in 2 Hz steps for phase and 15−400 Hz in 5 Hz steps for amplitude) with a simpler 2nd order Butterworth filter (4th order after application of the MATLAB “filtfilt” function) [7] instead of the EEGLAB-built filter. In contrast to the CM, the filter width of the phase frequencies was consistent with the steps of the phase frequency bins: 4 Hz. The filter width for the amplitude frequencies, however, was dependent on its comparison phase frequency, such that it was twice the compared center phase frequency. This was to assure that all appropriate side bands introduced by phase coupling were captured in the filtering process (see [7, 17, 20] for more details). The ranges and bandwidths can be adjusted with smaller phase bandwidths leading to PAC analyses with higher frequency resolution (S2B and S2E Fig).

#### Wavelet Method

The WM uses the same center frequencies as the methods above (4−52 Hz in 2 Hz steps for phase and 15−400 Hz in 5 Hz steps for amplitude) but instead of filtering, a convolution with Morlet wavelets is used. This wavelet’s cycles linearly progressed from 3−10 Hz as phase frequency increased to 50 Hz or as amplitude frequency increased to 400 Hz (see also [21]). Since the cycles of the wavelet are not dependent on the phase bandwidths, no change is seen when they are shortened (not shown). However, keeping the cycle size constant or changing the range of the cycles may lead to increases in frequency resolution depending on the coupling target (S2C and S2F Fig).

#### MI procedure

After the signals were appropriately filtered and binned (as described above), the resulting binned filtered signals ($x_{k}^{f}$) were Hilbert-transformed, resulting in their analytic signals, $y_{k}^{f}\left(t\right)$. This analytic signal is of the form

$$y_{k}^{f}\left(t\right)=x_{k}^{f}\left(t\right)+iH\left(x_{k}^{f}\left(t\right)\right),$$

where *H* is the Hilbert function. Using MATLAB’s angle function on each $y_{i}^{f_{\theta }}$, we obtained the phase of each signal, *θ*_*i*_. The amplitude of each signal, *A*_*j*_, was calculated as $⎸y_{j}^{f_{A}}⎸$. For each pair of *θ*_*i*_ and *A*_*j*_, we binned *θ*_*i*_ into 18 non-overlapping 20-degree bins, covering the entire 0−360° range, and calculated the mean amplitude in each bin k as ${\langle A_{j}\rangle }_{theta_{i}}\left(k\right)$. We then normalized each mean amplitude by dividing it by the sum of amplitudes over all bins (*N* = 18),

$${\langle {\bar{A}}_{j}\rangle }_{\theta_{i}}\left(k\right)\;=\;\frac{{\langle A_{j}\rangle }_{\theta_{i}}\left(k\right)}{\sum_{k=1}^{N}{\langle A_{j}\rangle }_{\theta_{i}}\left(k\right)}$$

Finally, the KL distance was obtained as

$$MI_{ij}=\frac{\text{log}\left(N\right)+\sum_{k=1}^{N}{\langle {\bar{A}}_{j}\rangle }_{\theta_{i}}\left(k\right)\text{log}\left({\langle {\bar{A}}_{j}\rangle }_{\theta_{i}}\right)}{\text{log}\left(N\right)}$$

*MI*_*ij*_ scores resulting from this procedure are high if the distribution of mean amplitudes differs substantially from a uniform distribution. Doing this process for every combination of *i* and *j*, we obtained a matrix of MI values for display in a phase-amplitude comodulogram, *Y*, in which each pixel (*MI*_*ij*_) is color-coded. To further quantify our data, we summed up specific rectangular regions of comodulograms (referred to as SumMI, see below). To further reduce noise, a surrogate analysis can be performed. Each surrogate consists of the original amplitude signal and a “shuffled” phase signal for which a PAC is computed [22]. To “shuffle” the signal while trying to retain some of the signal’s original structure, the original phase signal was split into 10 ms sections which were randomly permuted and concatenated. We used 100 surrogates. The means and standard deviations of the comodulograms based on these surrogates were used to z-score the original data. Results with this technique are shown in (S3–S5 Figs). However, because this method is computationally very expensive, we chose to not to use surrogates for the majority of the results that were based on synthetic data.

### Measurement of computational costs

Measurements of computational costs of calculating comodulogram were carried out using a series of synthetic signals with varying lengths. The measurements were done with MATLAB’s “tic” and “toc” routines. We report the median of measurements using five randomly chosen PAC targets for each of 199 different data lengths from 10 s to 1000 s for each variant of MI calculations (using the CM, VM or WM). For these calculations, we did not generate plots of comodulograms, in order to avoid inclusion of plotting runtime in our measurements.

### Automated detection of PAC peaks in comodulograms

We developed an algorithm to automatically detect PAC peaks in comodulograms. We focused this analysis on the accuracy of capturing multiple or changing targets during PAC capture, as we feel that this was one of the most important components of PAC analysis that could not be addressed through other technical solutions, such as increasing signal duration or lowering the SNR. For this analysis, the comodulogram matrix, *Y*, of all MIs (encompassing 25 phase frequency ranges and 78 amplitude frequency ranges) was sorted from high to low values, and a “matching threshold” was set as the median of the first 25 ⋅ 78 ⋅ 2^−*T*^ values, rounded down, with a value of *T* = 2.5 (found through optimization procedures described below). The highest MI value above this threshold, denoted *x*, was then selected as the center of the first (*N* = 1) “confidence region” and was assigned a confidence value, *c*(*x*, *N*). We define *c*(*x*, *N*) by constructing a sigmoid-like curve that accounts for the large range of possible MI values defined by the following equation:

$$c\left(x,N\right)=\frac{\max(\min(\frac{T_{u}-T_{\ell }}{{\text{log}}_{10}(\frac{u}{\ell })}\log_{10}(\frac{x}{\ell })+T_{\ell },1),0)}{N}$$

where, *u*, ℓ, *T*_*u*_, and *T*_ℓ_ define the empirically chosen thresholds of the curve, N is the current number of confidence regions, and 0 ≤ *c* ≤ 1. Using this equation MI values above *u* (chosen to be 10^−3^) are assigned a confidence value greater than *T*_*u*_ (chosen to be 0.9) while MI values lower than *l* (chosen to be 10^−5^), are assigned a confidence value below *T*_*ℓ*_ (chosen to be 0.1) We then added each adjacent pixel in the comodulogram to the confidence region if its value too was higher than the matching threshold. This process was then repeated for several rounds “growing” the region in each direction until adjacent pixels were either below the threshold or a maximum distance, *d*_max_ = 24 (found through optimization below), of pixels away from the center. These added pixels were given a confidence value based on their respective distance to the center, *d*, defined by: *D*(*d*, *x*, *N*) = *e*^−*d*/*δ*^ *c*(*x*, *N*) with *δ* = 6.2 (found through optimization, see below). We then removed all values of the thereby defined confidence region from the original sorted list of MI values, denoted the largest remaining MI (above the matching threshold) as *x*, and repeated the process for *N* = *N* + 1. Once the values above the matching threshold were exhausted, all remaining MI values, *x*_*i*_, were given a confidence value of 0.1 × *c*(*x*_*i*_, *N* + 1). To further evaluate the accuracy with which the matrix of confidence values, *C*, matched the (known) targets, we devised an error function (denoted “Match Score”) defined as $\frac{\#\text{targets}\;\text{hit}}{\#\text{targets}}⎸\text{Sum}(4C^{2}\circ Y)-\text{Sum}\left(Y\right)⎸$, where ∘ denotes element by element multiplication (Hadamard product). A target is considered a “hit” if a confidence region exists at the prescribed target location. Since *C*, the matrix of confidence value, has values ranging from 0 − 1, the 4*C*^2^ multiplier penalizes confidence values below 25% and favorably weights any confidence value over 50%. The values for the maximum distance, *d*_max_ = 24, confidence decay, *δ* = 6.2, and tolerance, *T* = 2.5, were calculated using a MATLAB algorithm (fmincon) to maximize the Match Score through 10 trials of dual random targets on all three calculation methods. Because the Match Score is influenced by the number of targets, the proximity of targets, and the resolution of the comodulogram, comparisons are only valid if the input signal is kept the same. We used the match score to compare the results of the three calculation methods, applied to identical input signals, making statistical comparisons between match scores when possible. Statistical comparisons of the three methods consisted of a one-way ANOVA followed by a two-sample t-test between the groups (if appropriate). A significance level of *α* = 0.001 was taken for the ANOVA and the two-sample t-tests to account for multiple comparisons.

## Results and discussion

### Influence of calculation method on runtime

**Fig 1:**
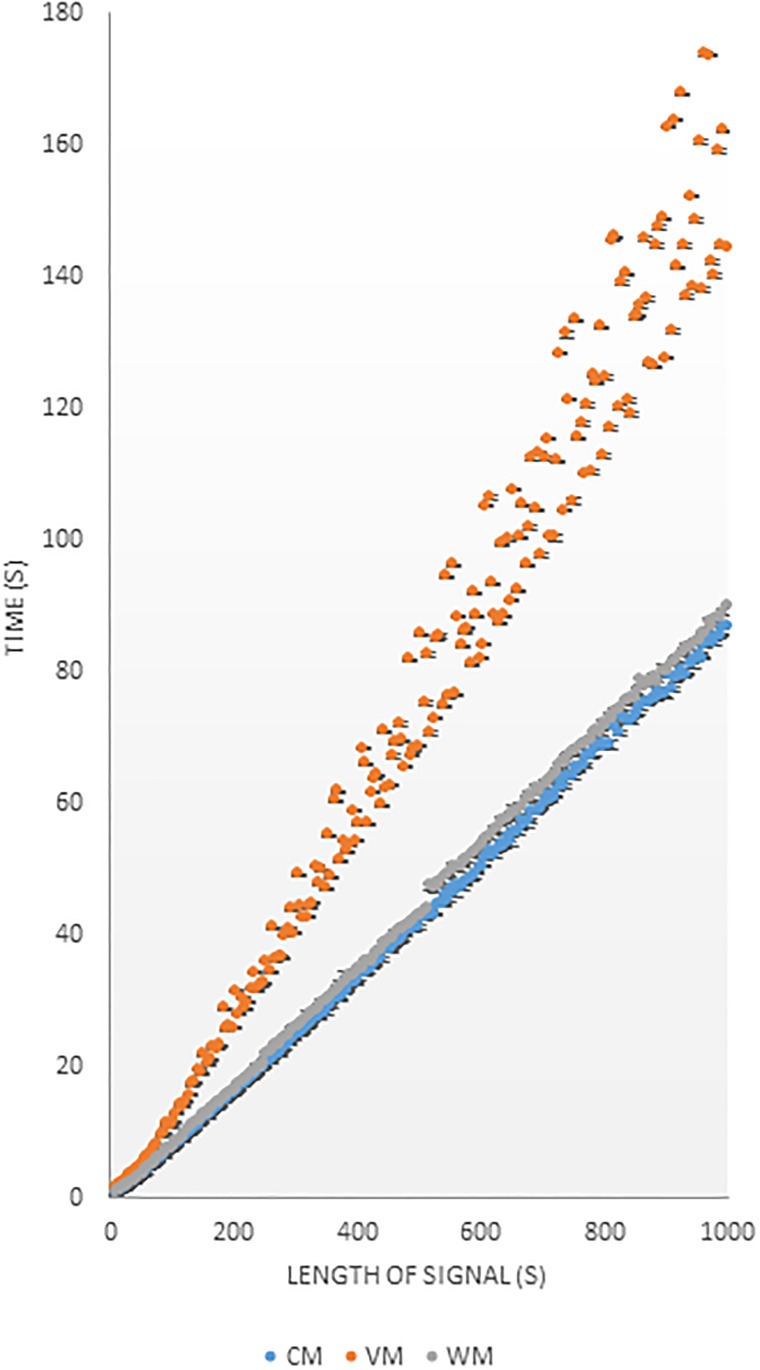
Computational Cost. Comparison of the computational costs of three filtering methods when calculating MI values for construction of comodulograms. Each data point represents the median of the amounts of time needed for PAC analysis of five random synthetic targets at a given signal length. Bars denote standard deviations. Signals were generated to present a sampling rate of 1000 Hz. Blue symbols, CM; orange symbols, VM; gray symbols, WM.

We measured the computer runtime needed to generate comodulograms with each of the three methods over a series of 199 epochs with varying lengths, each containing five randomly placed PAC targets (Fig 1).

Our analysis shows that the VM takes much longer than the other two methods. Even shorter time scales (< 20*s*) require almost twice the computational time when using the VM, compared to the CM or WM. The two high-cost bottlenecks in the current implementation of the code fall around the filtering procedure and the sorting into phase bins. All three methods use the same sorting procedure, so there should be no differences in complexity with regard to this procedure. The CM requires one filtered signal for each phase step and each amplitude step (say, *p* + *a* computations). The VM, on the other hand, requires one filtered signal for each phase step and one for every phase-amplitude combination (this would be: *p* + *p* × *a* computations, or (*p* − 1)*a* more computations than CM), explaining the lower efficiency of the VM algorithm. In our implementation of the WM, we take advantage of wavelets multiplicative properties for the WM, obviating the need for computing each pair separately. Because the VM is more computationally expensive, it may be advantageous to use CM or WM algorithms in time-sensitive applications.

### Influence of the length of the signal on PAC target detection

**Fig 2:**
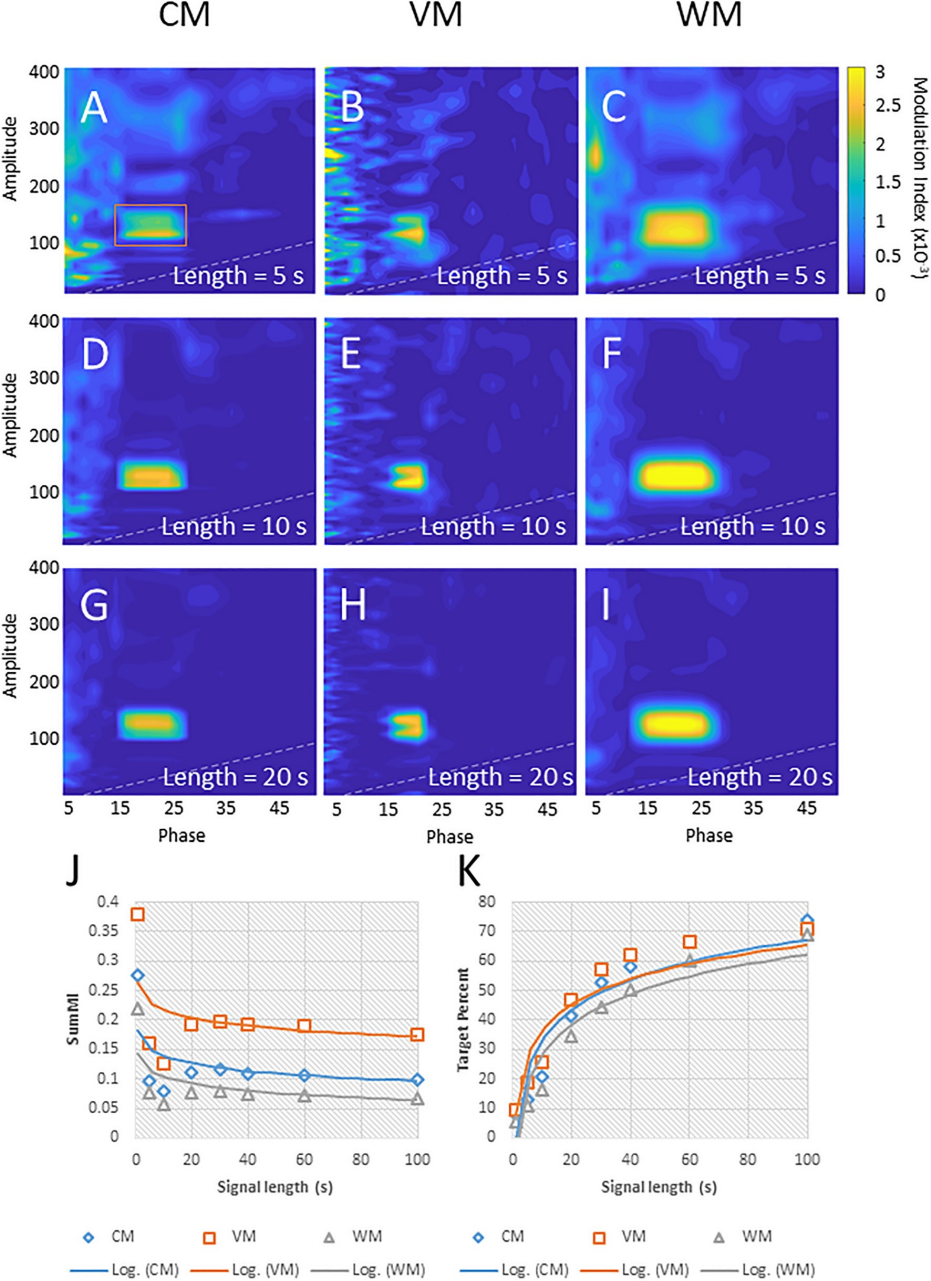
Effects of the duration of input signals on PAC measurements. A-I: comodulograms of signals with “target” PAC at 20 Hz (phase) and 130 Hz (amplitude), using the CM (A, D, G), VM (B, E, H), or WM (C, F, I), respectively, and signal lengths of 5 s (A-C), 10 s (D-F), and 20 s (G-I). J shows the sum of the modulation indices around the target (phase: 14−26 Hz; amplitude: 100−160 Hz; see red rectangle in A) and their logarithmic trend lines. K shows the percentage of PAC found in the red rectangle around the target over that of the entire comodulogram. In each comodulogram, the area below the white dashed line represents an area of low confidence (as frequencies for phase and amplitude are close together). All comodulograms are scaled to the same color range. The axis titles ‘Phase’ and ‘amplitude’ in A-I refer to frequencies, measured in Hz.

The length of the signal (number of samples) also influences the reliability of PAC target detection. Tort et al. found that longer signal lengths generally reduced MI variation but did not recommend a minimal signal length [15]. Published studies use a minimum length ranging from 15 s [7] to 30 s [2, 3, 9, 15, 18]. Fig 2 shows the results of using the CM, VM, and WM method to determine PAC peaks within the same synthetic signals, using a target combination of 20 Hz (phase) and 130 Hz (amplitude), with a stable signal-to-noise ratio. The first row (A-C) show comodulograms based on a 5 s signal, the second (D-F) on a 10 s signal, and the third (G-I) on a 20 s signal. As the length increases, the apparent noise in the comodulogram decreases and the PAC at the target becomes more clearly defined. To show the change in PAC sensitivity we calculated the SumMI value, as the sum of MI values within a range of values for phase and amplitude (14 − 26 Hz range for phase, 100 − 160 Hz range for amplitude; see red rectangle in Fig 2A) around the target PAC for varying lengths of the signal (Fig 2J). With all three methods, the SumMI values stabilized around a signal length of 20 s. We also calculated the ratio of the SumMI in the rectangle around the target and the sum of all MI values within the comodulogram (Fig 2K), as a measure of the reliability of PAC detection. The resulting values increased following a logarithmic trendline, demonstrating the value of choosing the longest possible data length for PAC analyses. In real-world applications, the signal length is obviously limited by the duration of artifact-free recordings and the biological stability of PAC. A time-resolved analysis of PAC (see below) may help to navigate the trade-offs of signal stability and accuracy of PAC determination in individual cases.

### Effect of signal to noise ratio on PAC detection

**Fig 3:**
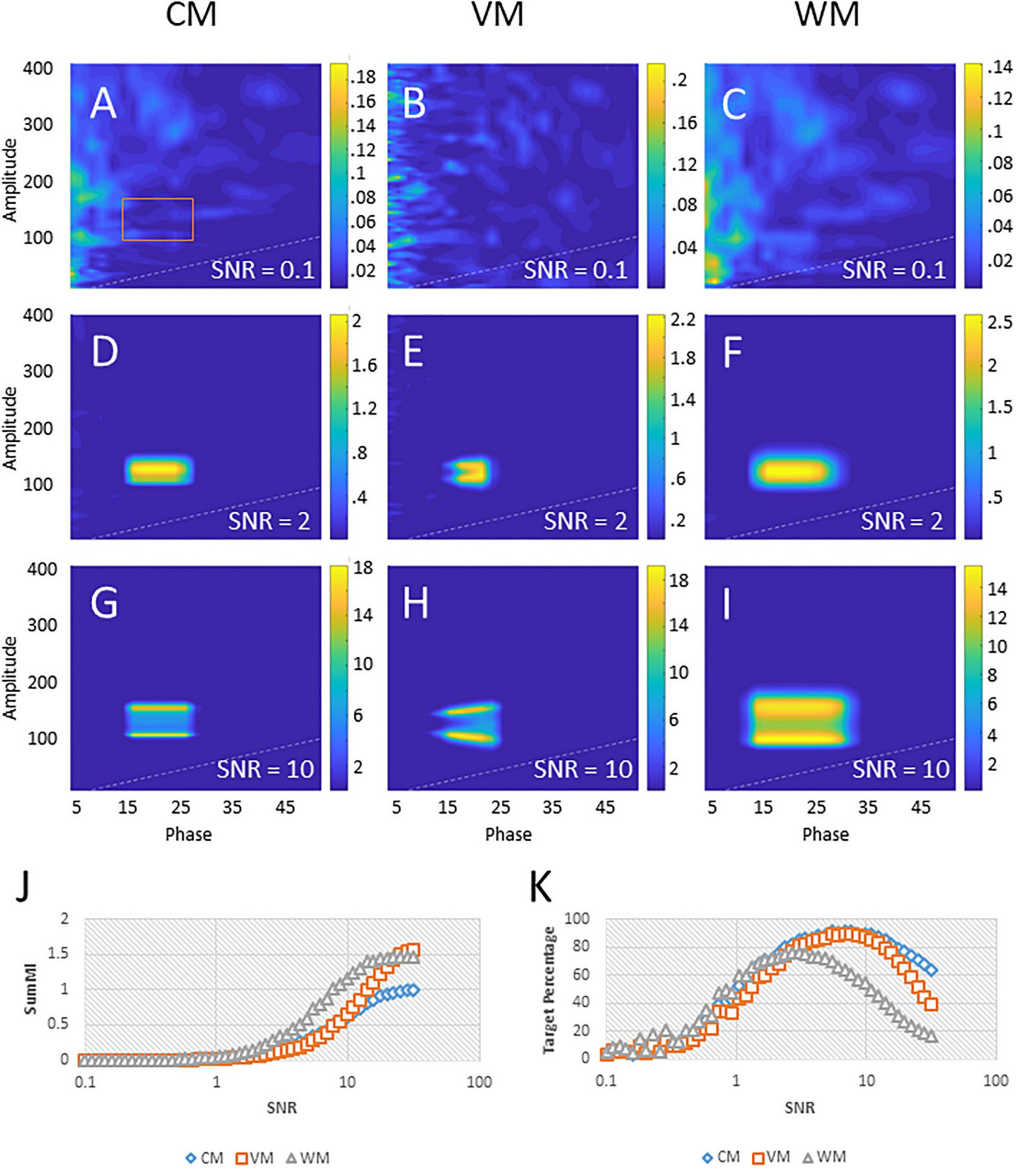
Effects of SNR on PAC measurements. The plots in A-I show example comodulograms computed for SNR = 0.1 (A-C), SNR = 2 (D-F), and SNR = 10 (G-I), with each column representing a different method (CM (A, D, G), VM (B, E, H), or WM (C, F, I), respectively). J shows a comparison of the SumMIs around the target (phase: 14−26 Hz range; amplitude: 100−160 Hz range; see red rectangle in A) and their logarithmic trendlines. K shows the proportion of the sum of PAC values in the rectangle around the target and the sum of all PAC values in the comodulogram.

To examine the effect of different levels of noise on PAC measurements, we generated PAC measures on the same 100 s synthetic signal using a target combination of 20 Hz (phase) and 130 Hz (amplitude), with different SNR values (see Fig 3). The PAC was calculated for all three methods (CM: A,D,G; VM: B,E,H; WM: C,F,I). We found that the most accurate PAC determination required the noise to be in a specific range. When the SNR was very small (SNR = 0.1), the target was not visible (A-C). When the noise was high (SNR = 10), the target was visible but broke up into subsegments (G-I) at amplitudes higher and lower than the target, likely because the filter width was too small to capture the side bands of the target (which therefore appeared as separate pseudo-targets). In the frequency domain, the filtering did not capture the sidebands in their entirely at SNR = 10; with the CM filter allowing for 50% capture and the VM allowing 70% capture. Fig 3J shows an increase in SumMI (the sum of MIs within the 14 − 26 Hz phase/100 − 160 Hz amplitude range, see red rectangle in A) as the SNR increases. The percentage of PAC in the region compared to the rest of comodulogram increases with small SNR values but decreases with higher ones (Fig 3K).

### Factors influencing the accuracy of PAC detection

**Fig 4:**
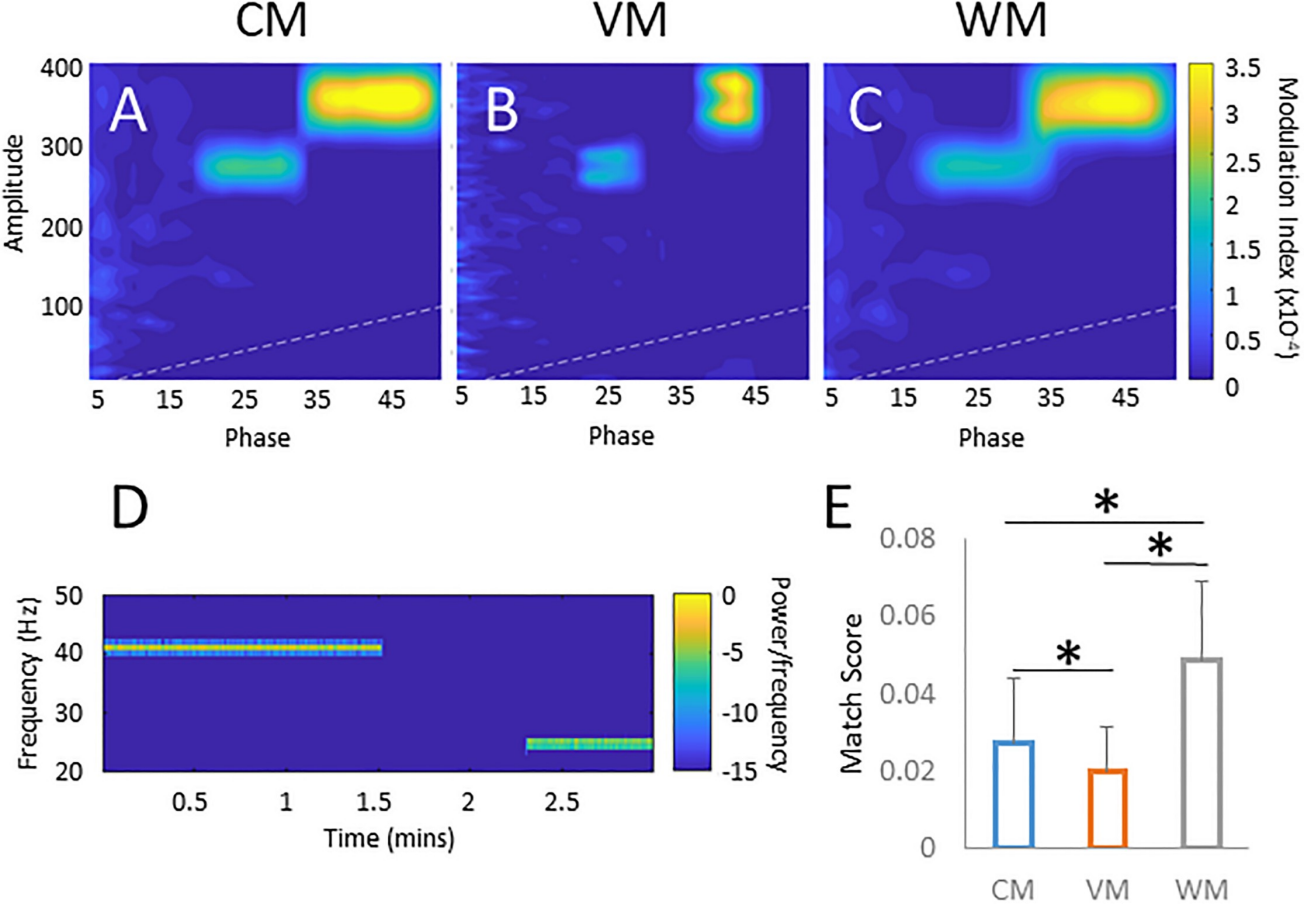
Effect of shifting targets on PAC measurements. The three comodulograms (A-C) show the same signal (D) presenting a randomly chosen target present for a random duration, followed by a gap of random length, and another randomly chosen target present for a random duration. E shows the match score of the three different methods over 500 trials. * denotes significance (*p* < .001) of a two-sample t-test after correction for multiple comparisons. All comodulograms are scaled to the same color range.

**Fig 5:**
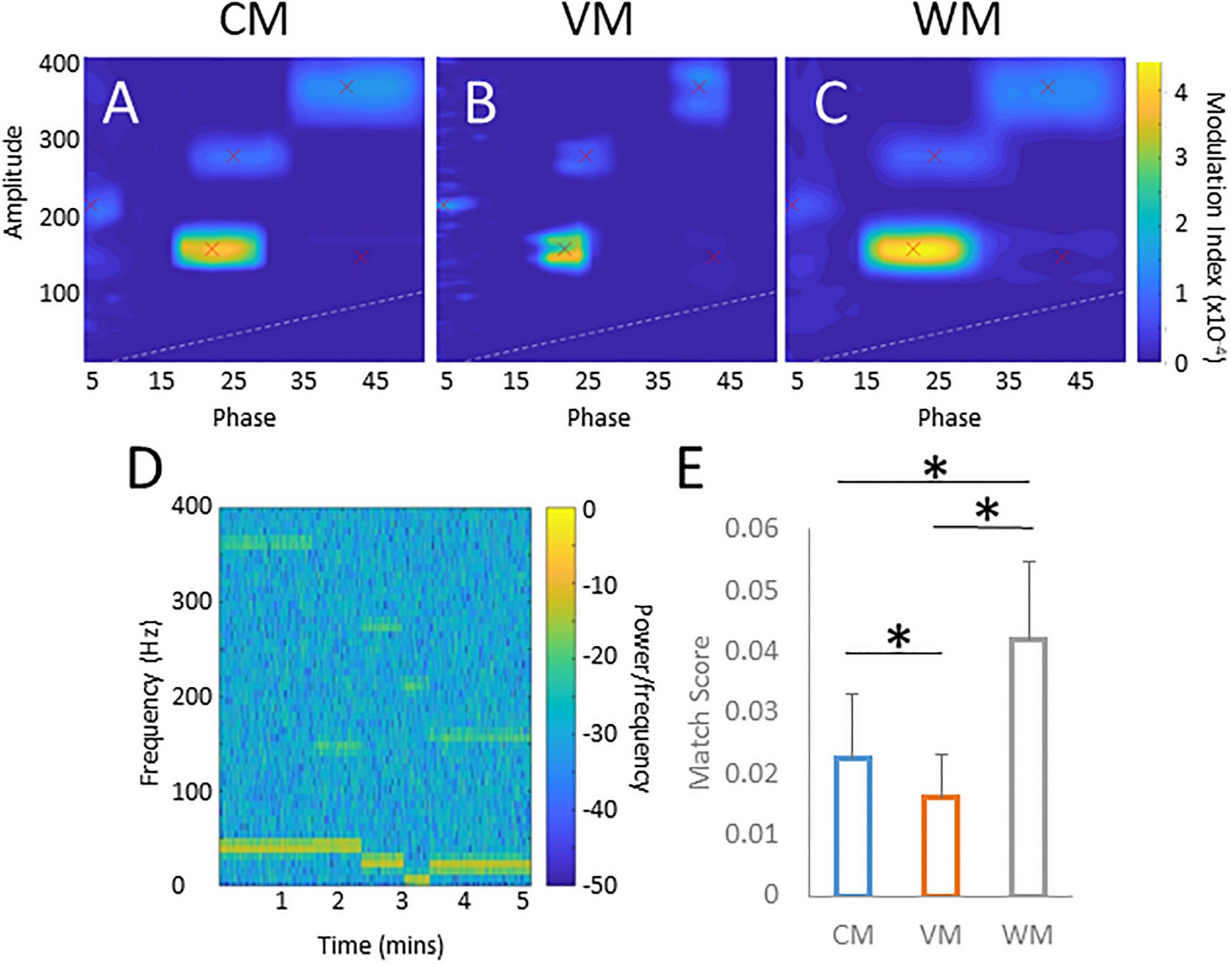
Effects of the presence of multiple targets on PAC measures. The three comodulograms (A-C) show examples of an analysis of a signal with five successively appearing random targets of varying lengths (signal shown in the spectrogram in (D)). E shows a Match score analysis (see methods) for 500 random trials. *, *p* < .001, two-sample t-test after correction for multiple comparisons. All comodulograms are scaled to the same color range.

In biological signals, PAC may be inconstant, or shift during the recording period. For instance, in cortical signals, factors such as ongoing behaviors, perception of stimuli, or the state of arousal may alter PAC [7, 9]. To demonstrate how the different filtering methods handle multiple or shifting targets, we constructed a signal with two random targets, separated by a gap (see Fig 4D for the spectrogram of the phase frequency). The images in Fig 4A–4C show comodulograms generated using the same signal by the three methods. While the amplitude frequency was similarly detected with all three methods, the VM provided better phase resolution than the two other methods. We used our target detection function (see Methods) to assess the accuracy of PAC identification. Using tests of 500 randomly “gapped” signals for each method, we found that the results differed significantly between the three methods (*p* < .001; one-way ANOVA), and that the WM performed significantly better than the other methods (*p* < .001; two-sample t-test after correction for multiple comparisons, see Fig 4E). We also created a synthetic signal containing five random PAC targets (see Fig 5D; spectrogram). The three methods were used to find PAC of this signal (Fig 5A–5C). The comodulograms rarely showed more than three targets. Match Scores were calculated for a set of 500 random 5-target sets (Fig 5E) and found significant differences between the methods (*p* < .001; one-way ANOVA). The WM performed better than the other methods (*p* < .001; two-sample t-test after Bonferroni correction).

### Time-resolved PAC Analysis

**Fig 6:**
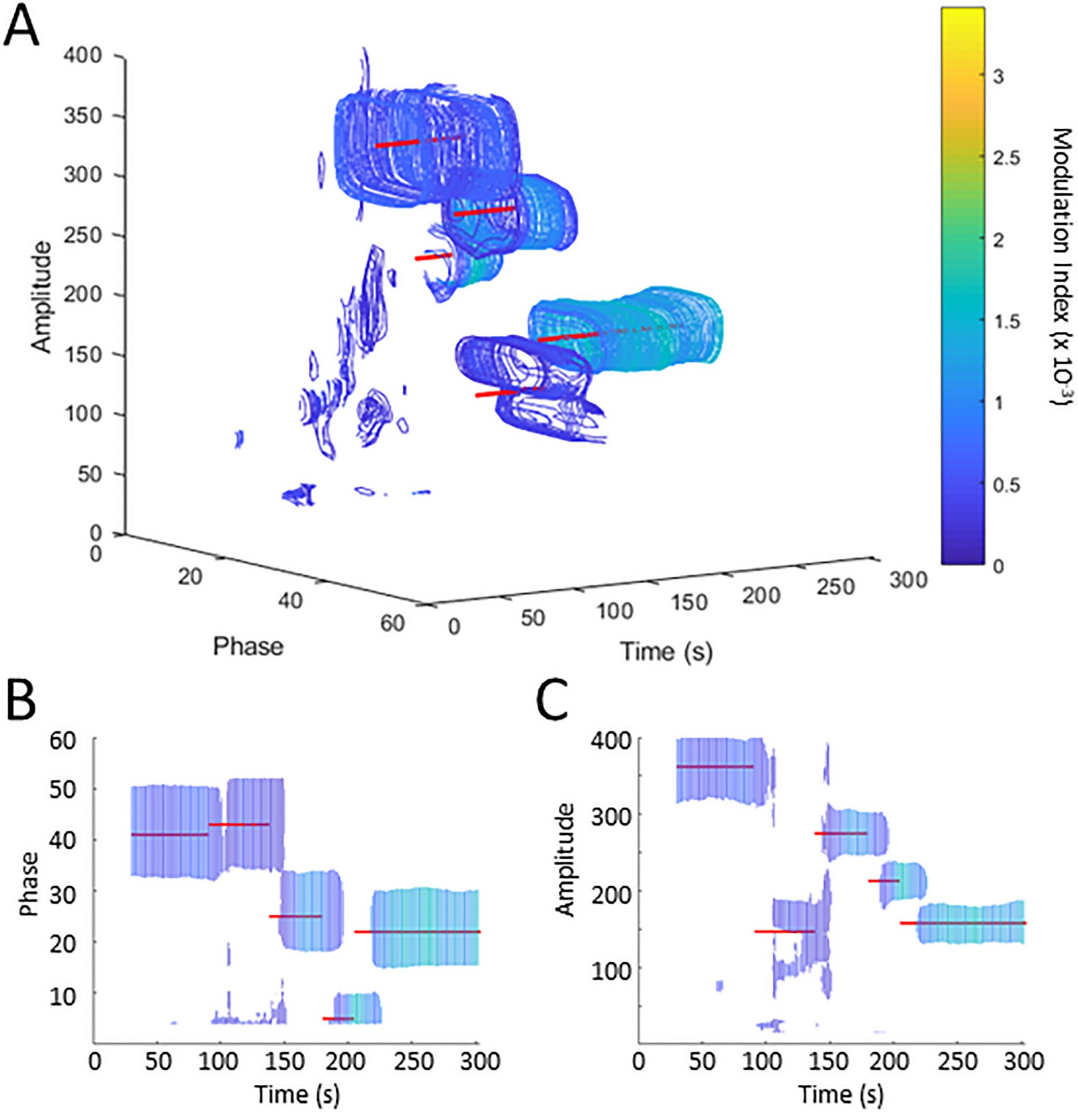
Time-resolved visualizations of PAC. Part A of the figure shows the time-resolved PAC for the signal in 5D, calculated by using the WM. B and C show the same 3D representation, projected onto the Phase-Time plane (B) or the Amplitude-Time plane (C). The red lines denote the synthetic targets. All comodulograms are scaled to the same color range.

**Fig 7:**
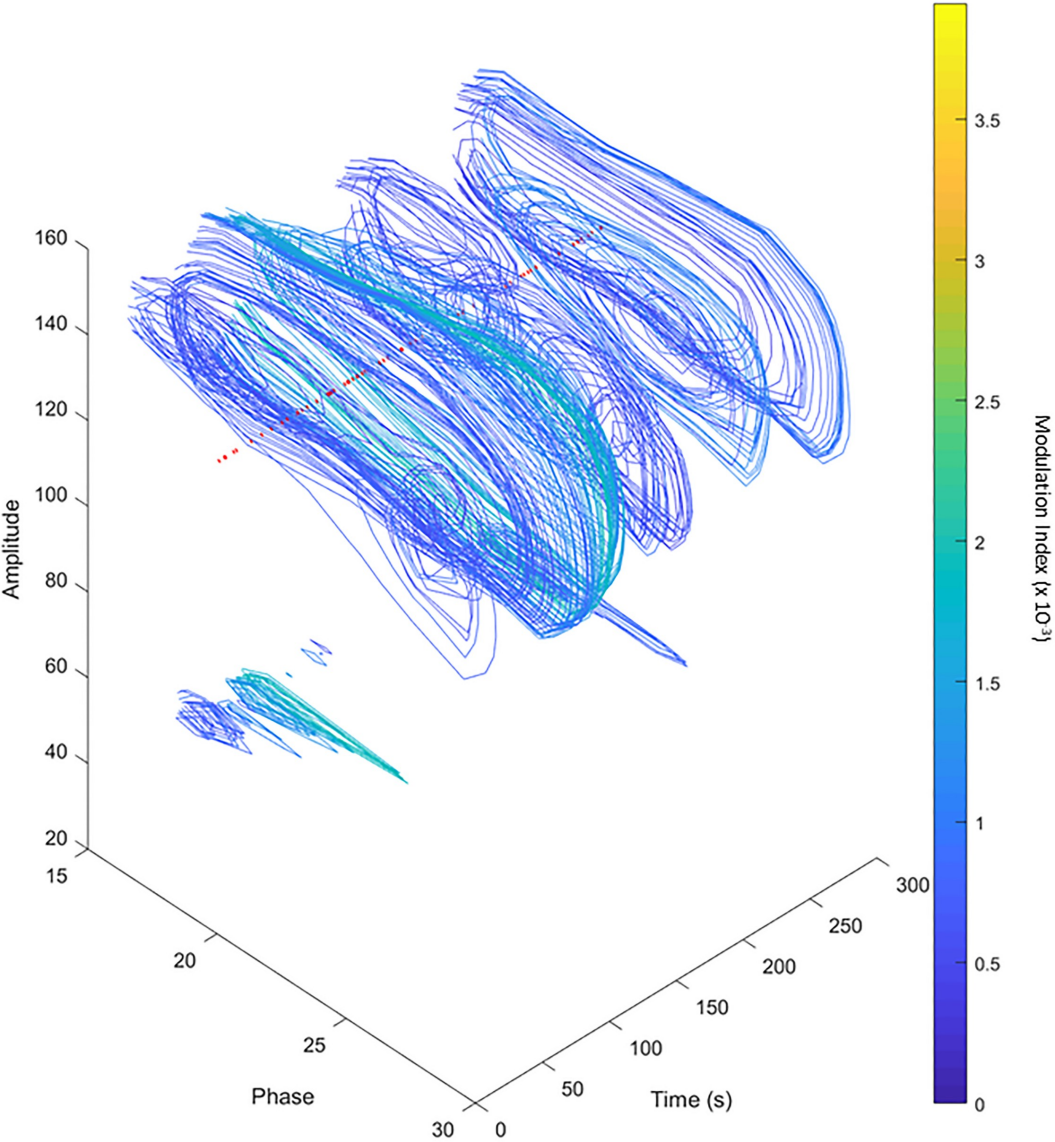
Time-resolved visualizations of dynamic PAC. Red lines indicate 1 s “burst” periods of 20 Hz (phase)/130 Hz (amplitude) PAC. Note that the phase axis is only shown for 15 − 30 Hz to better visualize the 3D structure.

Since short signal durations reduce the sensitivity and accuracy of PAC detection (Fig 2) while long signal durations with unstable PAC can lead to misidentification or missing PAC peaks (e.g., Figs 4 and 5), we investigated whether exploration of time-resolved PACs can be used to optimize the evaluation periods for given signals. By generating comodulograms of overlapping 30 s-long data segments, starting every second, we computed a time-resolved comodulogram as shown in Fig 6, using WM analyses applied to the signal shown in Fig 5D. In the pseudo-3D representation in Fig 6A, PAC is more easily identifiable than in 5C. Additionally, the three-dimensional comodulogram can be projected onto either the phase and amplitude planes (Fig 6B and 6C, respectively) to illustrate how phase and amplitude of the participating PAC components change through time. Fig 6 shows that the changes in coupling as time progresses match the intended targets (red lines). The apparent 30 s offset of the identified PAC peaks from the (known) appearances of the PAC targets (compare Fig 5D with Fig 6B/6C) results from our method of aligning the PAC measure to the original signal. Recent studies have shown that beta band oscillations in LFPs recorded in the basal ganglia in patients with Parkinson’s disease fluctuate in amplitude, with epochs of intense LFP beta-band activity lasting up to 1 s [23, 24]. To examine the usefulness of the time-resolved PAC analysis for such signals, we attempted to capture random 1 s bursts of synthetic signals (20 Hz phase/130 Hz amplitude) during a 5 min segment of white noise (Fig 7). We found that the time-resolved PAC captured many of these “bursts” (red lines), provided the SNR was high. Unfortunately for signals with shorter time scales such as those in perceptual/cognitive experiments, this may not always be the case. There is no clear way to avoid the minimum amount of time needed for analysis, as a sufficient amount of cycles of each filtered signal must be present. Thus, reliable ‘real-time’ PAC analyses, based on very short data segments cannot be done with this method. This obviously limits the value of the proposed time-resolved PAC method to cases in which long data segments are available. Comparing signals against phase shuffled surrogates may be able to extend the lower limit on signal length. S3 and S4 Figs show comodulograms generated with the CM, WM, and VM, used with or without z-scoring against surrogates on a 30 s signal and a 3 s signal, respectively. In S3 Fig, the surrogate analysis obviously reduces the noise around the actual target (20 Hz phase and 130 Hz amplitude), while in S4 Fig, the surrogate analysis reveals the signal, even though it is barely visible in the comodulograms based on the original data alone. Although the surrogate results in these examples are impressive, the use of surrogate analyses can also add noise to PAC analysis, making it difficult to locate relevant PAC in recorded data (S5 Fig). We recommend such time-resolved analyses as a starting step for off-line PAC analyses. Similar methods to examine PAC through time have been done in the past (e.g. PACograms [7]). However, they limit their analysis to two dimensions, losing or averaging the phase or amplitude planes. The three-dimensional display of PAC shown here will allow investigators to more easily account for changes in PAC itself, or for external changes that may affect PAC such as extraneous movement or drowsiness [7, 9, 21].

## Conclusion

The KL distance MI is considered to be the best current method for calculating PAC because it is noise-tolerant and amplitude-independent. Our studies show that several issues limit the interpretation of PAC analyses, including the duration of the underlying data stream, the SNR of the signal (which influences the detectability and shape of the detected PAC), and the method used to detect PAC. Given the sensitivity of the analysis to these factors, it is important to communicate not only the results of comodulogram analyses, but also to be explicit regarding the specifics of the data used, and the algorithm used for filtering the data. Additionally, initial time-resolved PAC analyses may help to determine some of the parameters to be used in subsequent conventional PAC analyses, specifically the most appropriate signal length which may differ between studies, dependent on signal stability. We found that the WM had overall the most favorable performance profile among the methods tested. This method has the additional advantage of providing the temporal features of the input signal (Figs 6 and 7), at a trivial computational cost.

## Supporting information

S1 Fig Example of an off-target peak detected by PAC analysis. This figure shows an example of the appearance of a false peak in PAC analysis. The result was seen when the target phase frequency was within 12 the amplitude frequency. For this example, the target phase frequency was set to 40 Hz and the target amplitude frequency to 60 Hz. The target is marked as a red x in these plots. The false peak was detected in comodulograms generated with the CM, WM, and the VM (top to bottom).
(TIF)

S2 Fig Effects of phase bandwidth on PAC. A, B: Effect of a 4 Hz, 2 Hz, 1 Hz phase bandwidth on an example synthetic LFP with low phase and low amplitude (marked as a red x) using both the CM (A) and VM (B). C: The figure shows comodulograms of the same synthetic signal, using the WM with cycle length progressing linearly from 3 − 10 Hz (top) and from 1.5 − 20 Hz (second from top), as well as with stationary cycle length of 3, 6.5, and 10 Hz (bottom three plots). D, E: Effect of a 4 Hz, 2 Hz, 1 Hz phase bandwidth on an example synthetic LFP with high phase and high amplitude (marked as a red x) using both the CM (A) and VM (B). C: PAC on the same synthetic LFP is shown using the WM with cycle lengths progressing linearly from 3 − 10 Hz and from 1.5 − 20 Hz (top plots), as well as with stationary cycle lengths of 3, 6.5, and 10 Hz (bottom three plots). Dotted lines are marked around the PAC computed with the 4Hz bandwidth (A, B, D, E) or the 3-10 Hz cycle length (C, F) for comparison.
(TIF)

S3 Fig Effects of surrogate analysis on extended signals. A 60-second section of data with a constant synthetic target at 20 Hz (phase) and 130 Hz (amplitude) was examined using the CM, WM, and the VM. The left column (colorbars indicate range of modulation indices) of plots shows the analysis of the original data. The right column shows the analysis of the data, z-scored to 100 phase-shuffled surrogates (colorbars indicate range of z-score ranges).
(TIF)

S4 Fig Effects of surrogate analysis on short signals. A 3-second section of data with a constant synthetic target at 20 Hz (phase) and 130 Hz (amplitude) was examined using the CM, WM, and the VM. The left column (colorbars indicate ranges of modulation indices) of plots show the analysis of the original data. The right column shows the analysis of the data, z-scored to 100 phase-shuffled surrogates (colorbars indicate z-score ranges).
(TIF)

S5 Fig Example of PAC analysis of a cortical LFP signal. A 60 s signal of M1 LFP activity recorded in a nonhuman primate was examined with the CM, WM, and VM (A). The same signal was then examined again after being z-scored to 100 phase-shuffled surrogates (B).
(TIF)
