# Supplementary figures and images for: Empirical analysis of phase-amplitude coupling approaches
Source: PLoS One. 2019 Jul 9;14(7):e0219264. doi: 10.1371/journal.pone.0219264 (PMC6615623; doi:10.1371/journal.pone.0219264)

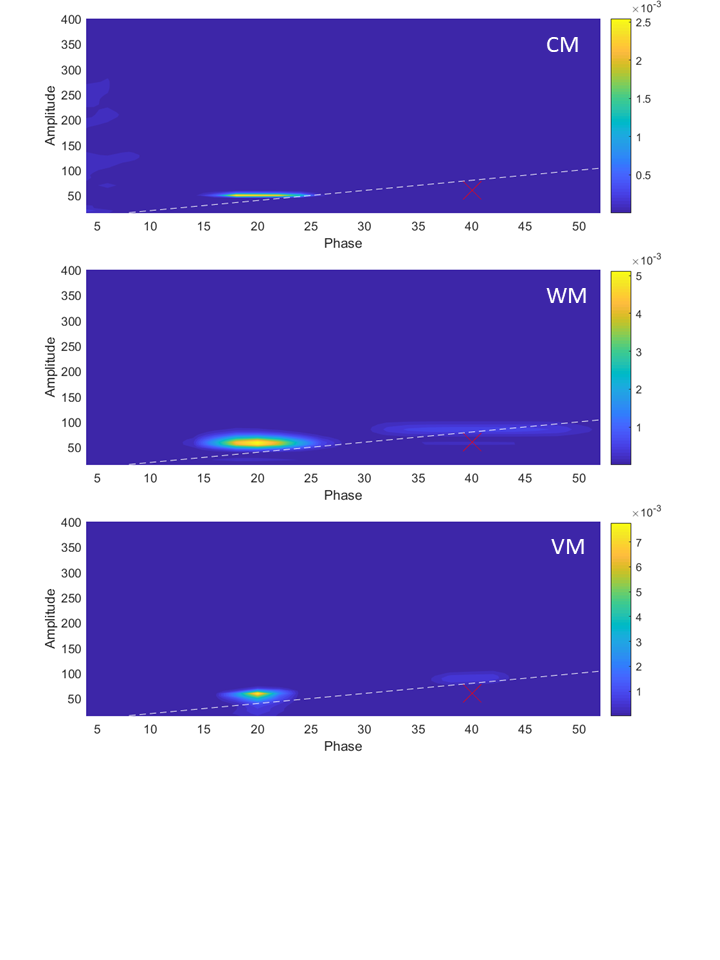

Supplement: S1 Fig — This figure shows an example of the appearance of a false peak in PAC analysis. The result was seen when the target phase frequency was within 12 the amplitude frequency. For this example, the target phase frequency was set to 40 Hz and the target amplitude frequency to 60 Hz. The target is marked as a red x in these plots. The false peak was detected in comodulograms generated with the CM, WM, and the VM (top to bottom). (TIF) [file pone.0219264.s001.TIF]

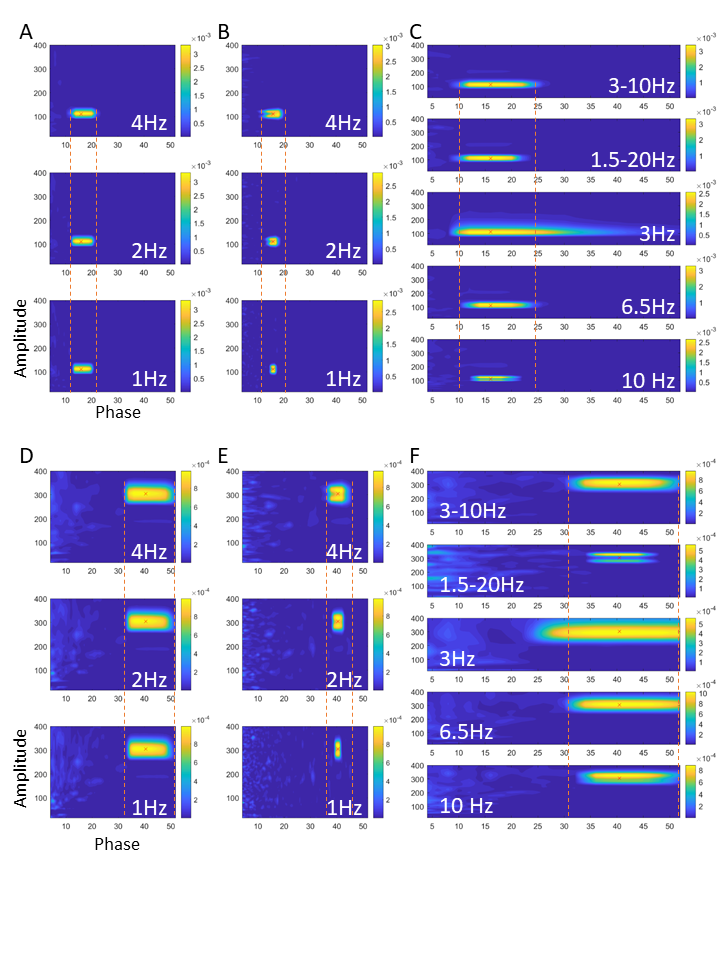

Supplement: S2 Fig — A, B: Effect of a 4 Hz, 2 Hz, 1 Hz phase bandwidth on an example synthetic LFP with low phase and low amplitude (marked as a red x) using both the CM (A) and VM (B). C: The figure shows comodulograms of the same synthetic signal, using the WM with cycle length progressing linearly from 3 − 10 Hz (top) and from 1.5 − 20 Hz (second from top), as well as with stationary cycle length of 3, 6.5, and 10 Hz (bottom three plots). D, E: Effect of a 4 Hz, 2 Hz, 1 Hz phase bandwidth on an example synthetic LFP with high phase and high amplitude (marked as a red x) using both the CM (A) and VM (B). C: PAC on the same synthetic LFP is shown using the WM with cycle lengths progressing linearly from 3 − 10 Hz and from 1.5 − 20 Hz (top plots), as well as with stationary cycle lengths of 3, 6.5, and 10 Hz (bottom three plots). Dotted lines are marked around the PAC computed with the 4Hz bandwidth (A, B, D, E) or the 3-10 Hz cycle length (C, F) for comparison. (TIF) [file pone.0219264.s002.TIF]

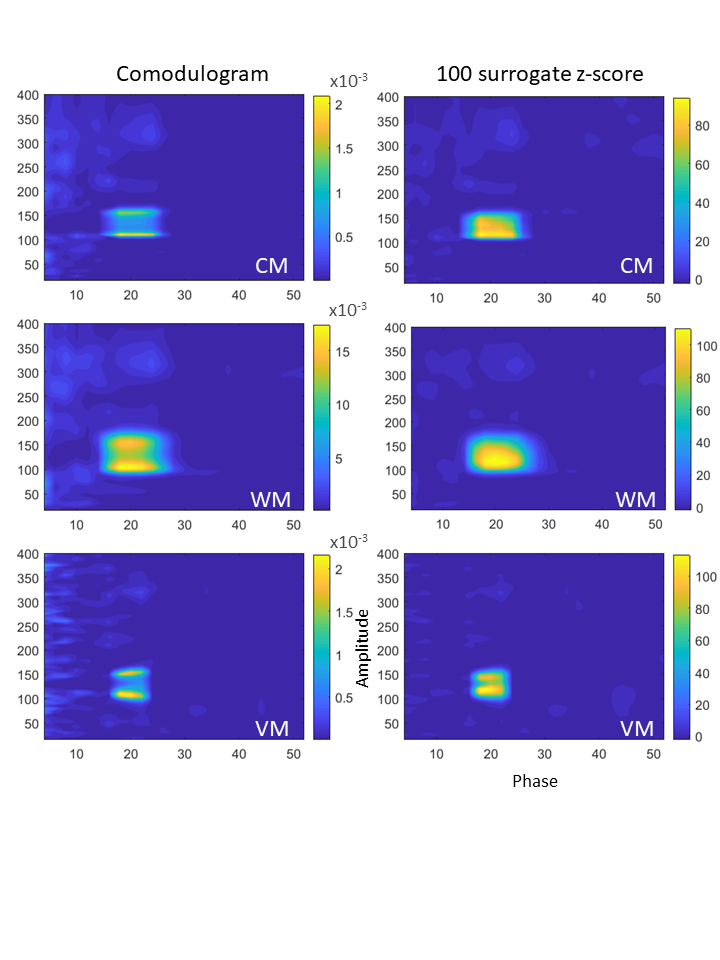

Supplement: S3 Fig — A 60-second section of data with a constant synthetic target at 20 Hz (phase) and 130 Hz (amplitude) was examined using the CM, WM, and the VM. The left column (colorbars indicate range of modulation indices) of plots shows the analysis of the original data. The right column shows the analysis of the data, z-scored to 100 phase-shuffled surrogates (colorbars indicate range of z-score ranges). (TIF) [file pone.0219264.s003.TIF]

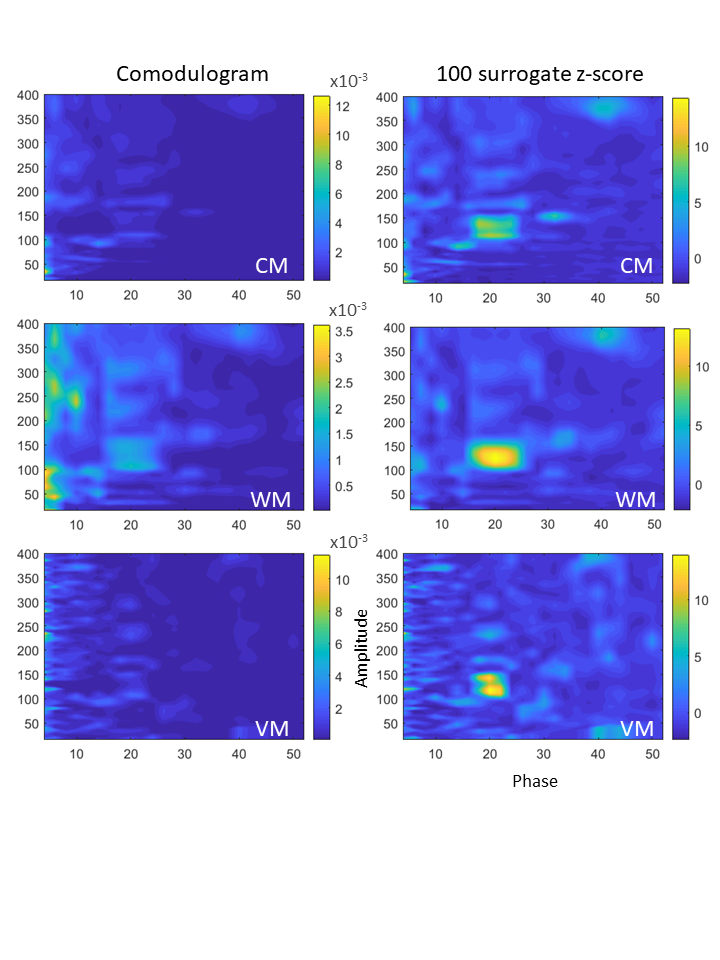

Supplement: S4 Fig — A 3-second section of data with a constant synthetic target at 20 Hz (phase) and 130 Hz (amplitude) was examined using the CM, WM, and the VM. The left column (colorbars indicate ranges of modulation indices) of plots show the analysis of the original data. The right column shows the analysis of the data, z-scored to 100 phase-shuffled surrogates (colorbars indicate z-score ranges). (TIF) [file pone.0219264.s004.TIF]

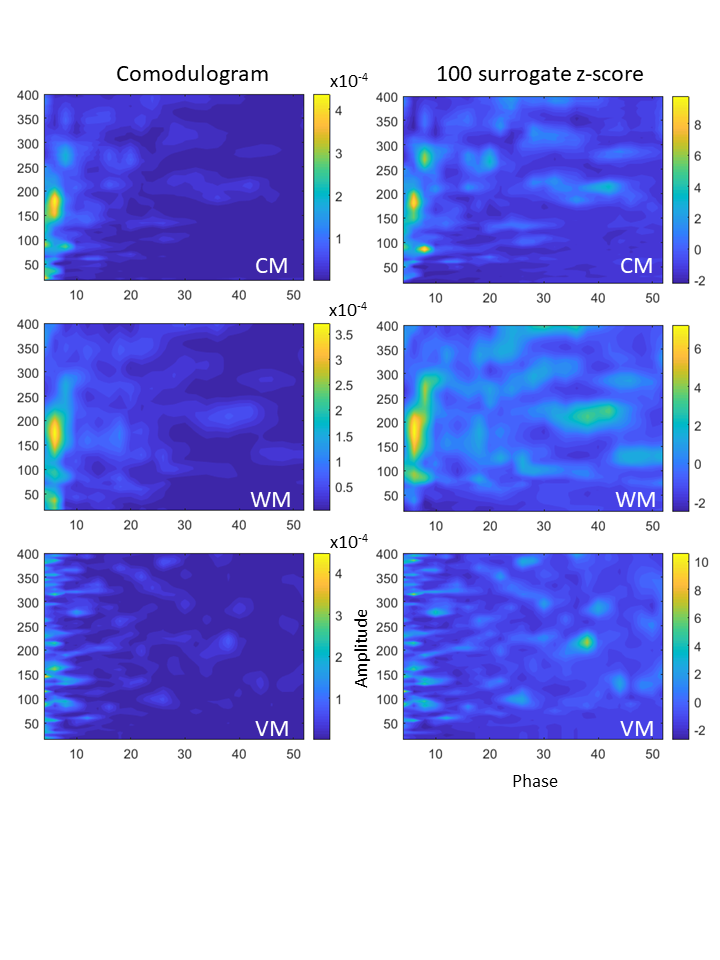

Supplement: S5 Fig — A 60 s signal of M1 LFP activity recorded in a nonhuman primate was examined with the CM, WM, and VM (A). The same signal was then examined again after being z-scored to 100 phase-shuffled surrogates (B). (TIF) [file pone.0219264.s005.TIF]
